# P-656. Epidemiology of Mycoplasma pneumoniae Infections after COVID-19 Pandemic

**DOI:** 10.1093/ofid/ofaf695.869

**Published:** 2026-01-11

**Authors:** Lakshmi Marimuthu, Heba Mostafa, Anna Sick-Samuels, David Villafuerte

**Affiliations:** Johns Hopkins University, Baltimore, Maryland; Johns Hopkins University School of Medicine, Baltimore, Maryland; Johns Hopkins University, Baltimore, Maryland; 3Johns Hopkins, Baltimore, Maryland

## Abstract

**Background:**

*M. pneumoniae* infections declined during the SARS-COV-2 pandemic and resurged in 2023. This study aimed to characterize contemporary clinical characteristics and macrolide- resistance of M.pneumoniae infections from 2023-2024 in a Maryland-based hospital system.

**Table 1:**
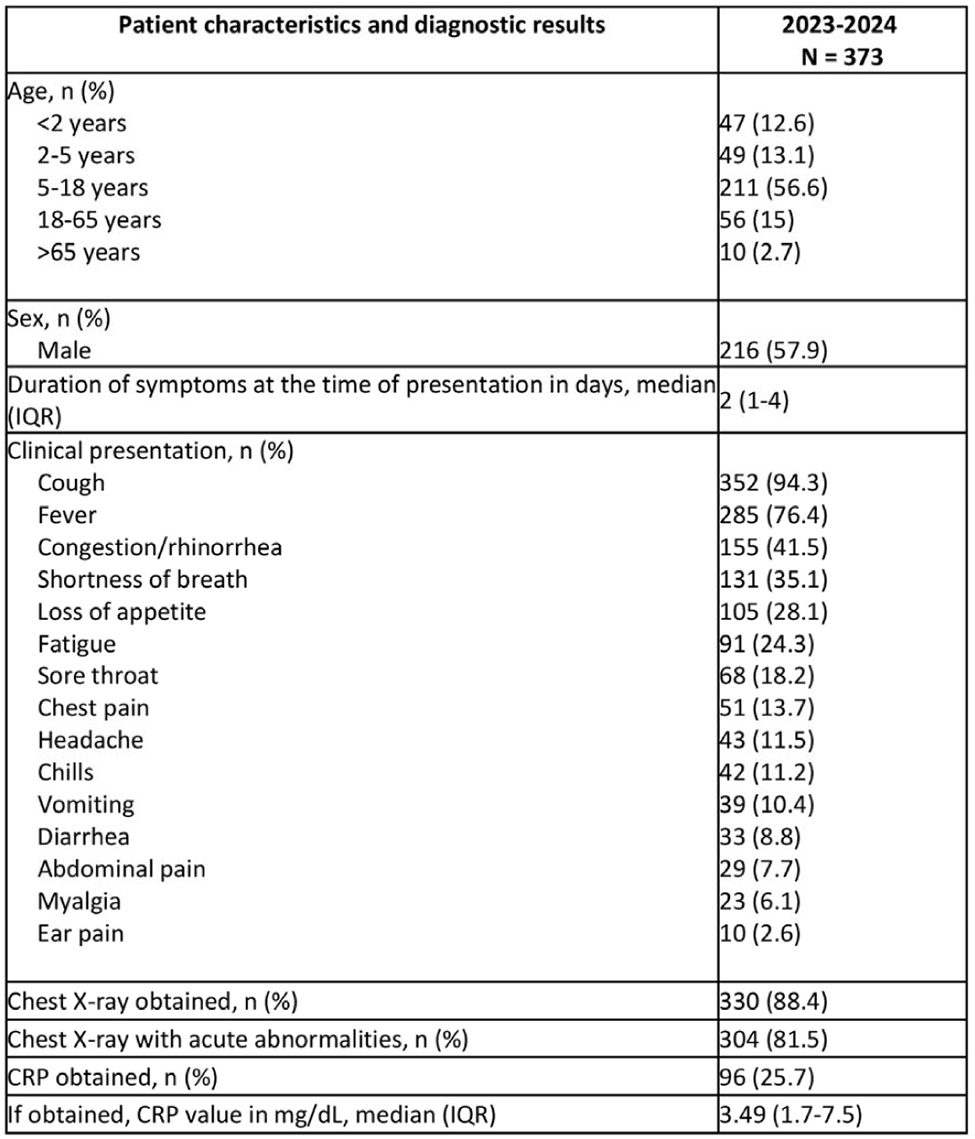
Characteristics and diagnostic results of patients with acute respiratory infection/pneumonia Abbreviations: IQR: Interquartile Range, CRP: C-reactive protein

**Table 2:**
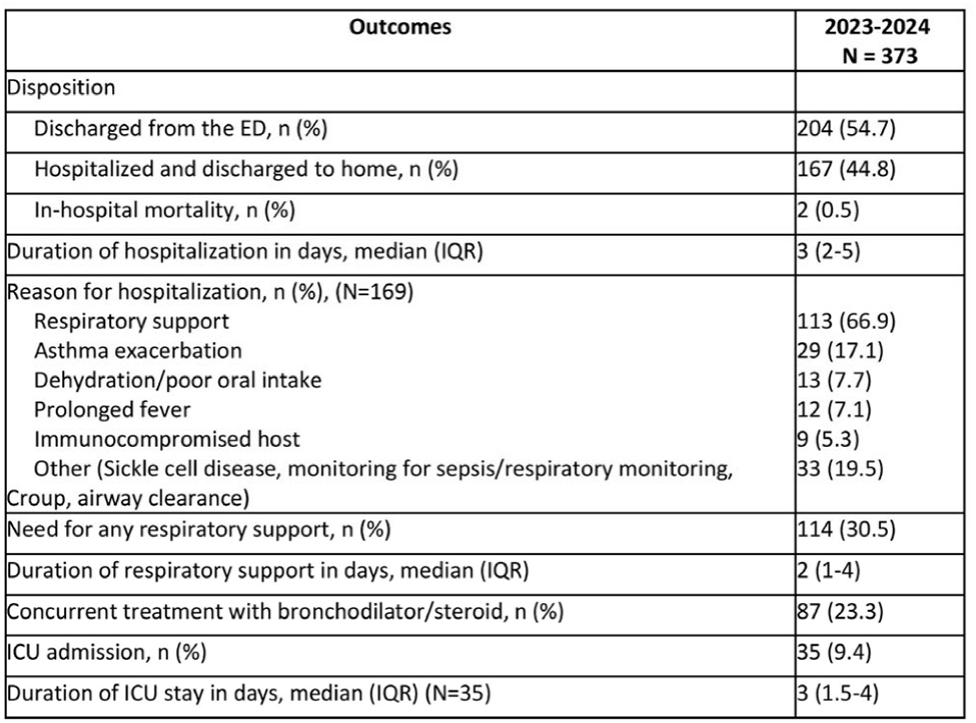
Clinical outcomes of patients with acute respiratory infection/pneumonia Abbreviations: ED: Emergency Department, ICU: Intensive Care Unit, IQR: Interquartile Range

**Methods:**

We conducted a retrospective cohort study of patients of all ages with *M.pneumoniae* infection presenting to 5 hospitals in the Johns Hopkins Health System in Maryland from 2023-2024. We reviewed the Electronic Health Record (EHR) for patients with positive Respiratory Pathogen Panel (RPP), IgM test result, or with *M.pneumoniae* ICD-10 codes and documented external test result. Patients lacking consistent clinical symptoms or with alternative diagnosis were excluded.  For resistance testing, *M.pneumoniae* DNA was extracted and amplified from RPP swabs. Library preparation was performed with nanopore sequencing and binary alignment maps were analyzed using Integrative Genomics Viewer 2.19.1.

**Results:**

We identified 431 patients, 377 (87.5 %) by RPP and 54 (12.5%) by IgM. In total, 373 (86.5 %) patients had signs of acute respiratory infection (Table 1). Of those, 25.7% were < 5 years, 56.6% were 5-18 years, and 17.7 % were adults. Most commonly, patients had cough, fever, and congestion, and 85% had abnormal Chest X-Ray (CXR) findings. Regarding outcomes (Table 2), 44.8% patients were hospitalized, 30.5% required respiratory support, and 9.4% had Intensive Care Unit (ICU) admission. We identified 58 patients with extrapulmonary manifestations with a median age of 10 years, 65.6% had dermatologic and 24.1% had central nervous system manifestations. We tested 134 (35.9%) RPPs for macrolide resistance testing, of which 35 (26.1%) had genetic polymorphism: 18 in A2063G, 4 in A2064G, and 13 in both genes.

**Conclusion:**

In this cohort, roughly one quarter of infections were in children < 5 years which is atypical for *M.pneumoniae* infections and the majority of patients had acute lung findings. Of tested specimens, 26% had polymorphism in macrolide-resistance genes. Next, we will assess clinical outcomes among those with detected macrolide resistance. These findings may inform future empiric treatment for *M. pneumoniae* infections.

**Disclosures:**

Heba Mostafa, MD, PhD, D(ABMM), Hologic, Qiagen, Diasorin: Grant/Research Support|Qiagen, Diasorin, Roche: Honoraria|Seegene: Advisor/Consultant

